# Expression Profile Screening and Bioinformatics Analysis of circRNA, LncRNA, and mRNA in Acute Myeloid Leukemia Drug-Resistant Cells

**DOI:** 10.4274/tjh.galenos.2019.2019.0312

**Published:** 2020-05-06

**Authors:** Meiling Li, Fuxue Meng, Quanyi Lu

**Affiliations:** 1 Zhongshan Hospital Affiliated to Xiamen University, Department of Hematology, Xiamen, China; 2 The Third Affiliated Hospital of Guizhou Medical University, Department of Hematology and Rheumatology, Duyun, China

**Keywords:** Acute myeloid leukemia, Drug resistance, CircRNA, LncRNA, Bioinformatics analysis

## Abstract

**Objective:**

Acute myeloid leukemia (AML) is a highly heterogeneous hematological malignancy, and drug resistance and relapse are key factors in the failure of leukemia treatment. Studies have increasingly shown that circRNA and LncRNA play important roles in the development of tumors, but their roles remain unclear in the mechanism of AML resistance.

**Materials and Methods:**

Resistant AML cell line HL-60/ADM (adriamycin, ADM) was constructed and circRNA, LncRNA, and mRNA expression profiles were screened followed by high-throughput sequencing. Bioinformatics analysis was then carried out, and the circRNA-miRNA ceRNA network was constructed and confirmed using qRT-PCR analysis.

**Results:**

A total of 1824 circRNAs, 2414 LncRNAs, and 5346 mRNAs were screened for differentially expressed genes. Enrichment analysis was performed utilizing Gene Ontology and the Kyoto Encyclopedia of Genes and Genomes, which mainly involved protein domain specific binding, transforming growth factor-β (TGF-β) receptor, and cellular metabolism. The mTOR signaling pathway, MAPK signaling pathway, RAP1 signaling pathway, and Akt signaling pathway were closely related to drug resistance.

**Conclusion:**

Our study provides a systematic outlook on the potential function of ncRNA in the molecular mechanisms of resistant AML cells. Hsa-circ-0000978 and hsa-circ-0000483 might serve as potential prognostic biomarkers and therapeutic targets of AML resistance.

## Abstract (tr)

**Amaç:**

Akut myeloid lösemi (AML) oldukça heterojen bir hematolojik malignitedir, ve tedavi başarısızlığında ilaç direnci ve nüks anahtar rol oynamaktadır. Çalışmalar, circRNA ve lncRNA’nın tümör gelişiminde önemli rol oynadığını artarak göstermektedir, ancak AML direnç mekanizmasında rolleri belirsizliğini korumaktadır.

**Gereç ve Yöntemler:**

Dirençli AML hücre hattı HL-60/ADM (adriamisin, ADM) oluşturuldu ve circRNA, LncRNA, ve mRNA ekspresyon profilleri yüksek-kapasitede dizileme sonrası tarandı. Sonra biyoinformatik analiz gerçekleştirildi ve circRNA-miRNA ceRNA ağı oluşturuldu ve qRT-PCR analizi kullanılarak doğrulandı.

**Bulgular:**

Farklı ifade edilen genler için toplam 1824 circRNA, 2414 LncRNA, ve 5346 mRNA tarandı. Başlıca protein domain spesifik bağlama, transforme edici büyüme faktörü-β (TGF-β) reseptörü, ve hücresel metabolizma ile ilgili ‘Gene Ontology’ ve ‘Kyoto Encyclopedia of Genes and Genome’ kullanılarak zenginleştirme analizi gerçekleştirildi. mTOR sinyal yolağı, MAPK sinyal yolağı, RAP1 sinyal yolağı ve Akt sinyal yolağı ilaç direnci ile yakından ilişkili idi.

**Sonuç:**

Çalışmamız, dirençli AML hücrelerinin moleküler mekanizmasında ncRNA’ın potansiyel fonksiyonuna sistematik bir bakış açısı sağlamıştır. Hsa-circ-0000978 ve hsa-circ-0000483 potansiyel prognostik biyogöstergeler ve AML direncinin terapötik hedefleri olarak işlev görebilirler.

## Introduction

Acute myeloid leukemia (AML) is a highly heterogeneous hematological malignancy. Although its treatment has made significant progress, the prognosis is still unsatisfactory. Recurrence and drug resistance are the main factors [1]. At present, there are many studies on the molecular mechanisms of AML resistance [2,3]. However, with the development of bioinformatics, the epigenetic mechanism in the pathogenesis of AML still remains unclear.

Among human transcripts, about 10%-20% are protein-encoding RNA, and the remaining 80%-90% are noncoding RNAs (ncRNAs) [4,5]. Long noncoding RNAs (LncRNAs) are a class of noncoding RNAs that regulate gene expression at the transcriptional or posttranscriptional level [6]. LncRNA plays an important regulatory role in the drug resistance process. Li et al. [7] reported that the LncRNA HOTTIP can promote the development of pancreatic cancer and regulate gemcitabine resistance by regulating HOXA13, while HOTTIP regulates cisplatin resistance in osteosarcoma cells by activating the Wnt/β-catenin pathway [8]. In addition, Qu et al. [9] found that in sunitinib-resistant renal cell carcinoma, when FOX0 and AKT expression decreased, LncRNA increased, knocking out LncRNA and then reversing drug resistance. Endogenous competition between mir-34 and mir-449 promotes the expression of AXL and c-MET in sunitinib-resistant renal cell carcinoma to regulate the drug resistance process, confirming that LncRNA can be used as a target to repair drug resistance. Circular RNAs (circRNAs) are novel noncoding RNAs characterized by a covalently closed structure with nonrandom spiking and RNase degradation resistance [10,11]. CircRNA is present in the cytoplasm and is extremely abundant and highly conserved and stable in the blood [12]. CircRNAs are increasingly found in various diseases and show cell or tissue specificity [13,14,15].

At present, the molecular mechanism of LncRNA and circRNA in resistant AML cells remains unclear. In this study, high-throughput sequencing of HL-60 and HL-60/ADM (adriamycin, ADM) was performed utilizing Gene Ontology (GO) and the Kyoto Encyclopedia of Genes and Genomes (KEGG). A circRNA-miRNA ceRNA network was constructed to provide new therapeutic targets and the theoretical basis for treatment of drug resistance in AML.

## Materials and Methods

### Materials

HL-60 cells were donated by Professor Lu Quanyi of the Key Laboratory of Hematology, Xiamen University, Xiamen, China. Basic RPMI 1640 Medium (GIBCO, Carlsbad, CA, USA), fetal bovine serum (FBS; ScienCell, Carlsbad, CA, USA), adriamycin (Haizhenghuirui Pharmaceutical Co. Ltd., Fuyang, Zhejiang, China), the Cell Counting Kit-8 (Dojindo, Tokyo, Japan), RIPA buffer (Beijing Solarbio Science & Technology Co. Ltd., Beijing, China), the BCA Protein Assay Kit and One-Step Western Kit HRP (Beijing Kangwei Century Biotechnology Co. Ltd., Beijing, China), GAPDH monoclonal antibody (ImmunoWay Biotechnology Co, Plano, TX, USA); P-gp monoclonal antibody (Abcam, Cambridge, MA, USA), and Immobilon Western Chemiluminescent HRP Substrate (Millipore Corp., Billerica, MA, USA) were also obtained.

## Methods

### Cell Culture and Drug Resistance Induction

HL-60 cells were incubated in basic RPMI 1640 Medium containing 10% FBS, 100 µg/mL streptomycin, and 100 U/mL penicillin at 37 °C and 5% CO_2_ under saturated humidity conditions after recovery. The liquid was changed once every 2 days, and 10^6^ cells/mL were amplified at a 1:3 ratio. ADM induction was performed in HL-60 cells by combining the concentration gradient increasing method and the impact method (high-dose intermittent induction) as referenced in the literature [16]. The initial induction concentration was 0.1 µg/mL, shock induction was performed for 1 h, and culturing was continued until HL-60 cells grew and proliferated normally at 1 µg/mL ADM. It took 8 months to successfully induce ADM resistance in HL-60 cells.

### CCK-8 Assay and Cell IC_50_ Values

HL-60 cells were collected and centrifuged at 1000 rpm/min for 5 min, and then they were resuspended in RPMI 1640 Medium and counted. Furthermore, 10^4^ cells of cell suspensions of 100 µL were placed in 96-well plates at 37 °C in a 5% CO_2_ incubator for culturing for 24 h. ADM was added with differences in concentrations of 10 µL and cells were incubated for 24 h, and then 10 µL of CCK-8 solution was added to each well. Culture plates were further incubated in the incubator for 4 h. OD values were measured and data were collected to calculate IC_50_ values, or ADM concentrations required for 50% inhibition in vitro.

### Western Blot Detection of the Expression of Drug-Resistant Protein

RIPA buffer was added with phenylmethanesulfonyl fluoride to collect the cells showing logarithmic growth, and proteins were extracted from the cells. Protein concentration was determined using the BCA Protein Assay Kit. Protein samples containing sample buffer were denatured for 5 min in boiling water. SDS-PAGE electrophoresis was performed with 25 µg of sample in each hole with the addition of 5 µL of prestained protein marker. When the bromophenol blue dye ran off the gel layer, the electrophoresis was terminated, and further experiments were performed on a 35 mA transmembrane overnight. The One-Step Western Kit HRP was used according to the manufacturer’s instructions. Rabbit P-gp antibody, antibody pretreatment solution, and dilution buffer solution were added, mixed, and poured onto the membrane. Immobilon Western Chemiluminescent HRP Substrate was used for color reaction.

### RNA Library Construction and Sequencing

High-throughput sequencing service was provided by CloudSeq Biotech (Shanghai, China). Transcriptome high-throughput sequencing and subsequent bioinformatics analyses were also performed by CloudSeq Biotech (Shanghai, China). Briefly, total RNA was used to remove the rRNAs using the Ribo-Zero rRNA Removal Kit (Illumina, USA) according to the manufacturer’s instructions. RNA libraries were constructed using rRNA-depleted RNAs with the TruSeq Stranded Total RNA Library Prep Kit (Illumina, USA) according to the manufacturer’s instructions. Libraries were checked for quality, and they were quantified using the Bioanalyzer 2100 system (Agilent Technologies, USA). Furthermore, 10 pM libraries were denatured as single-stranded DNA molecules, captured on Illumina flow cells, amplified in situ as clusters, and finally sequenced for 150 cycles on an Illumina HiSeq Sequencer according to the manufacturer’s instructions.

### Bioinformatics Analysis

For circRNA, high-quality reads were aligned to the reference genome/transcriptome with STAR software (v2.5.1b) and circRNAs were detected and identified with DCC software (v0.4.4). edgeR software (v3.16.5) was used to normalize the data and perform analysis of differentially expressed circRNAs. GO and KEGG analyses were performed for the differentially expressed circRNA-associated genes.

For LncRNA and mRNA, high-quality reads were aligned to the human reference genome (UCSC hg19) with HISAT2 software (v2.0.4). Then, guided by the Ensembl gtf gene annotation file, the Cuffdiff program (v2.2.1, part of Cufflinks software) was used to get the FPKM (fragments per kilobase of exon model per million reads mapped) for the expression profiles of LncRNA and mRNA. Accordingly, fold change and p-values were calculated based on FPKM, and differentially expressed LncRNAs and mRNAs were identified. LncRNA target genes were predicted by locations in relation to nearby genes, and GO and pathway analyses were performed on these target genes.

### Construction of circRNA-miRNA ceRNA Network

CircRNA-miRNA interactions were predicted using miRcode (http://www.mircode.org/) and TargetScan (http://www.targetscan.org/vert_72/) based on seed-match sequences. The circRNA-miRNA network was then constructed using Cytoscape software (http://www.cytoscape.org/).

### Validation of Differentially Expressed circRNAs

Total RNA was extracted by TRIzol (Invitrogen Life Technologies, Shanghai, China) to synthesize cDNA via reverse transcription. Quantitative real-time PCR was performed on the ViiA 7 Real-Time PCR System (Applied Biosystems) using the qPCR SYBR Green Master Mix (CloudSeq). Primer design was as follows: chr13: 50054355-50057699+ (F 5’-CCTGAATCCAAGACAGCCA-3’, R 5’-AAGGGGGAAGTTTTGGCA-3’), chr18: 21644104-21649235+ (F 5’-GAAAATCCGCCCCCTCTA-3’, R 5’-TGACAAAGCTGGCTCCAA-3’), chr7: 22330794-22357656- (F 5’-CATTCCTGCCAGAGGTGG-3’, R 5’-TGGGAAGGCGTATGTTCAA-3’), chr2: 15629018-15651474- (F 5’-CATCTGGGCGATTCCATC-3’, R 5’- AACCCCGTCTCCACCATT-3’), and ACTB (F5’-GTGGCCGAGGACTTTGATTG-3’, R 5’-CCTGTAACAACGCATCTCATATT-3’). The target RNA and internal parameters of each sample were subjected to real-time PCR, which was repeated three times. The data were analyzed by the 2^-ΔΔC^_T_ method.

### Statistical Analysis

All of the experimental data are presented as mean ± standard deviation (SD), and the t-test was used for comparisons between the two groups. Values of p<0.05 were considered to be significantly different. GraphPad Prism 5 software was used for statistical analysis.

## Results

### Construction of HL-60/ADM Drug-Resistant Cell Lines

**Figure 1:**
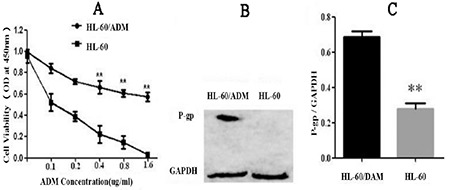
Detection of HL-60/ADM resistance: A) HL-60/ADM IC_50_ test, where the ADM concentration gradient was set at 0, 0.1, 0.2, 0.4, 0.8, and 1.6 μg/mL. The horizontal axis is ADM concentration and vertical axis is inhibition rate. B) Expression of drug-resistance related protein P-gp. C) P-gp/GAPDH ratio (**p<0.01).

Adriamycin resistance was induced in HL-60 cells via a combination of the concentration gradient method and the impact method (high-dose intermittent induction method) according to the literature [16], and it took 8 months to obtain HL-60/ADM resistant cell lines. IC_50_ detection is shown in Figure 1A. The expression of the drug resistance protein P-gp in HL-60/ADM resistant cells was significantly higher than that in HL-60 cells, with significance at p<0.01 (Figures 1B and 1C).

### Expression of circRNA, LncRNA, and mRNA in Resistant Cells

**Figure 2:**
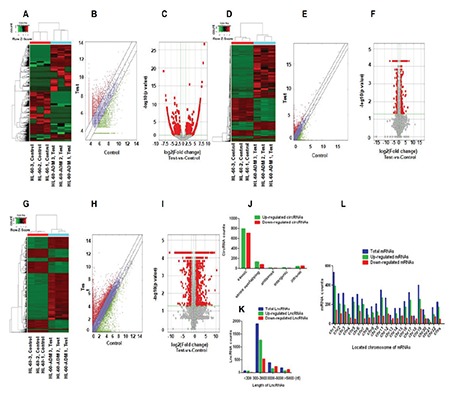
Differential expression of circRNA, LncRNA, and mRNA in HL-60/ADM. A-C) Hierarchical clustering, scatter plots, and volcano plots of the differentially expressed circRNAs in HL-60 and HL-60/ADM, respectively. D-F) Hierarchical clustering, scatter plots, and volcano plots of the differentially expressed LncRNAs in HL-60 and HL-60/ADM, respectively. G-I) Hierarchical clustering, scatter plots, and volcano plots of the differentially expressed mRNAs in HL-60 and HL-60/ADM, respectively. J) The catalog of differentially expressed circRNAs. K) Distribution of differentially expressed LncRNAs based on the length of nuclear acids. L) Distribution of differentially expressed mRNAs based on the location on human chromosomes.

To analyze the gene expression of resistant AML cells, we performed high-throughput sequencing of circRNA, LncRNA, and mRNA, and we screened differentially expressed genes. Hierarchical cluster analysis showed that the expression patterns of drug-resistant and drug-sensitive cells were significantly different (circRNA, Figure 2A; LncRNA, Figure 2D; mRNA, Figure 2G). Scatter plots were used to evaluate circRNA between drug-resistant and drug-sensitive cells. LncRNA and mRNA signal values were normalized to log2 values for visualization of expression differences (Figures 2B, 2E, and 2H, respectively). Volcanic maps were constructed based on fold change (FC≥1.2) and p-value (<0.05), and volcano maps of the differentially expressed genes between these two different conditions are provided in Figures 2C, 2F, and 2I. The general characteristics of RNA include RNA type, length, and localization distribution, as shown in Figures 2J-2L. The results showed that resistant AML cells differentially expressed 1824 circRNAs, 2414 LncRNAs, and 5346 mRNAs.

### Functional Analysis of Differentially Expressed circRNA, LncRNA, and mRNA

**Figure 3:**
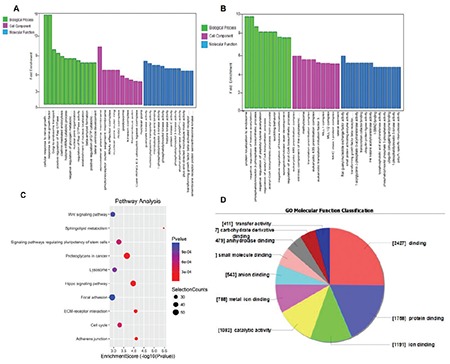
GO and KEGG pathway analysis of circRNA, LncRNA, and mRNA. A-B) Analysis of GO in terms of upregulation and downregulation of circRNA. C) Pathway analysis of upregulation of LncRNA. D) GO molecular function classification for upregulation of mRNA.

To explore the underlying genomics mechanisms involved in the developmental disorders of AML tumorigenesis, GO and KEGG pathway enrichment analyses of differentially expressed genes were used to evaluate candidate RNA functions. The GO terms with the highest enrichment scores for upregulated circRNA targeting were ribonucleoside triphosphate catabolic process and purine ribonucleoside triphosphate catabolic process (BP), intracellular (CC), and adenyl ribonucleotide binding (MF); for LncRNA, the GO terms involved macromolecule modification (BP), cornified envelope (CC), and protein homodimerization activity (MF); and anatomical structure morphogenesis (BP), cytoplasm (CC), and protein binding (MF) belonged to the GO analysis of mRNA. Furthermore, KEGG pathway analysis was performed to predict potential module functions. The KEGG analysis results were as follows: for circRNA, the B cell receptor signaling pathway (hsa04662), T cell receptor signaling pathway (hsa04660), MAPK signaling pathway (hsa04010), and mTOR signaling pathway (hsa04150); for LncRNA, signaling pathways regulating pluripotency of stem cells (hsa04550); and for mRNA, the Wnt signaling pathway (hsa04310), Rap1 signaling pathway (hsa04015), p53 signaling pathway (hsa04115), and VEGF signaling pathway (hsa04370). These are closely related to cancer progression and are significantly enriched in AML (Figure 3).

### Construction of circRNA-miRNA ceRNA Network

**Figure 4:**
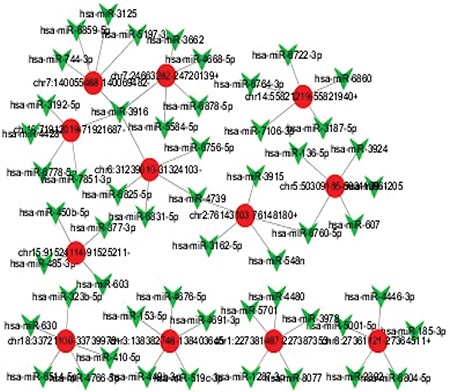
Network of twelve differentially expressed circRNA and miRNA genes predicted in drug-resistant cells. CircRNA: Red circles; MiRNA: green polygons. Twelve different genes were selected from the upregulated circRNAs to construct the circRNA-targeted miRNA gene network. Each circRNA is shown with five miRNA predicted targets.

To fully understand the underlying mechanisms of circRNA and AML development, based on differentially expressed circRNA data, we used a database to predict target miRNAs interacting with circRNA, and Cytoscape was used to construct a circRNA-targeted miRNA gene network map (Figure 4). For a particular miRNA, circRNA has many targets, and the network map illustrates the first five predicted miRNA targets that differentially express circRNA.

### Validation of circRNA Expression by RT-qPCR

**Figure 5:**
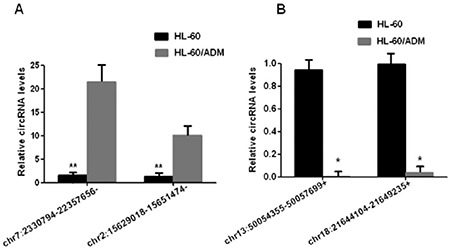
Expression levels of differentially expressed circRNA were detected by RT-qPCR. The horizontal axis is genes and the vertical axis is circRNA expression levels. A) Two genes, chr7:2330794-22357656- and chr2:15629018-15651474, were selected from circRNA with upregulated expression. B) chr13:50054355-50057699+ and chr18:21644104-21649235+ were selected from downregulated circRNAs. The target RNA and internal parameters of each sample were subjected to real-time PCR, which was repeated three times. The data were analyzed by 2-^ΔΔC^_T_ method. **p<0.01,*p<0.05.

Two upregulated genes (chr7: 2330794-22357656- and chr2: 15629018-15651474-) and two downregulated genes (chr13: 50054355-50057699+ and chr18: 21644104-21649235+) were selected from the circRNAs with differential expression. Differentially expressed circRNA levels were verified by RT-qPCR. As shown in Figure 5, the results of four circRNAs were consistent with the trend observed in circRNA sequencing.

## Discussion

At present, chemotherapy is still one of the main treatments for leukemia. However, multidrug resistance and treatment are key factors in the failure of leukemia treatment. Several factors are involved in the mechanism of leukemia resistance, including ABC transporter-mediated multidrug resistance [17], DNA repair abnormalities [18], variations in the bone marrow microenvironment [19], and abnormal expression of noncoding RNAs including circRNA, miRNA, and LncRNA [20]. Undeniably, ncRNAs have opened up new prospects for AML diagnosis, prognosis, and treatment. Indeed, the expression of specific ncRNAs such as circRNAs and LncRNAs could assist clinicians in classifying subtypes, evaluating prognosis, and predicting the response to drug treatment in AML. Garzon et al. [21] evaluated the associations of LncRNA expression with clinical characteristics, gene mutations, and outcomes and constructed an LncRNA score including 48 LncRNAs for independently predicting outcome prognosis, confirming that LncRNAs can assist in predicting clinical outcomes in older patients with CN-AML. Moreover, Li and Sun [22] reported that SNHG5 overexpression was frequently observed in AML patients with advanced FAB classification and unfavorable cytogenetics. Furthermore, a higher SNHG5 expression level was also associated with shorter overall survival. However, comprehensive analyses of the profiles of differentially expressed circRNAs, LncRNAs, and mRNAs in resistant AML cells have not been studied. Thus, we explored the expression profiles and predicted the potential functions of circRNAs, LncRNAs, and mRNAs in resistant AML cells by utilizing RNA high-throughput sequencing and bioinformatics analysis.

The numbers of differentially expressed genes of circRNAs, LncRNAs, and mRNA in resistant AML cells are 1824, 2414, and 5346, respectively. GO and KEGG pathway analyses of differentially expressed LncRNAs mainly revealed protein domain specific binding and protein dimerization activity. This provides a basis for important contributions to the development and resistance of leukemia. Although the understanding of the nature and function of circRNAs is still limited, it is undeniable that circRNA has always been a research hotspot in the field of ncRNAs, which particularly regulate miRNA-targeted gene expression as ceRNA molecules [23,24]. GO and KEGG pathway analyses predicted that these differentially expressed circRNA functions were related to tumor development, drug-resistant regulation, and metabolism-related pathways.

GO analysis mainly revealed involvement with PI3K activity, transforming growth factor-β (TGF-β) receptor, and cellular metabolism. Sui et al. [25] reported that activation of the PI3K/Akt/NF-κB pathway promotes P-gp expression, and the inhibition of this pathway reverses P-gp-mediated multidrug resistance. Zhou et al. explored the effect of the PI3K-specific inhibitor ZSTK474 on K562/A02 cells and their results showed that ZSTK474 reversed the resistance of K562/A02 cells to ADM and imatinib by downregulating P-gp expression; accordingly, the target of ZSTK474 for CML treatment is PI3K [26]. It can be seen that PI3K activity plays an important role in the regulation of drug resistance in leukemia. Results of KEGG analysis indicated that the upregulated circRNA in drug-resistant cells was mainly related to the mTOR signaling pathway, MAPK signaling pathway, and Akt signaling pathway. The PI3K/Akt signaling pathway maintains a close relationship between tumor cell multidrug resistance and P-gp. Studies have shown that activation of the PI3K/Akt signaling pathway increases drug efflux via the ATP-binding cassette (ABC) transporter [27], while the blocking of PI3K/Akt signaling pathways leads to downregulation of P-gp and MRP1 expression, restoring sensitivity to chemotherapeutic drugs [28].

According to the ceRNA theory, a circRNA-miRNA regulation network is present in cases of resistant AML. Moreover, differentially expressed circRNAs-miRNA interactions were predicted and the potential molecular mechanisms were further explored. Among these predicted potential target miRNAs, hsa-miR-24-2-59 is reported to be upregulated during hematopoietic cell terminal differentiation, suppressing MYC expression [29]. Hsa-miR-181b-5p may play a prominent role in pituitary adenoma as an effective biomarker and therapeutic target [30]. However, there are some limitations to this study, such as a small sample size and the in vitro research being conducted only on HL-60 cells. The next step of this work will be to verify the expression of LncRNAs and circRNAs in AML patients and to study the mechanisms of LncRNAs and circRNAs in the development of resistant AML.

## Conclusion

Even though only resistance to adriamycin was assessed in this study, the results suggest that the expression changes of circRNA/LncRNA regulate the cell resistance of AML, providing a new theoretical basis for the further understanding of multidrug resistance mechanisms and targeted therapies in AML.
